# *Megamonas funiformis,* Plasma Zonulin, and Sodium Intake Affect C3 Complement Levels in Inactive Systemic Lupus Erythematosus

**DOI:** 10.3390/nu15081999

**Published:** 2023-04-21

**Authors:** Bianca Depieri Balmant, Danielle Cristina Fonseca, Ana Paula Aguiar Prudêncio, Ilanna Marques Rocha, Letícia Callado, Juliana Tepedino Martins Alves, Raquel Susana Matos de Miranda Torrinhas, Eduardo Ferreira Borba, Dan Linetzky Waitzberg

**Affiliations:** 1 Laboratory of Nutrition and Metabolic Surgery of the Digestive System, LIM 35, Department of Gastroenterology, Hospital das Clínicas HCFMUSP, Faculdade de Medicina, Universidade de São Paulo, São Paulo 01246-903, Brazil; 2 Hospital Sírio Libanês, Department of Medical Clinical Nutrition, Brasilia 70200-730, Brazil; 3 Rheumatology Division, Hospital das Clínicas HCFMUSP, Faculdade de Medicina, Universidade de São Paulo, São Paulo 01246-903, Brazil

**Keywords:** inactive lupus disease, food intake, sodium intake, intestinal permeability, gut microbiota, inflammation

## Abstract

The etiology of systemic lupus erythematosus (SLE) remains unclear, with both genetic and environmental factors potentially contributing. This study aimed to explore the relationship among gut microbiota (GM), intestinal permeability, and food intake with inflammatory markers in inactive SLE patients. A total of 22 women with inactive SLE and 20 healthy volunteers were enrolled, and dietary intake was assessed through 24-h dietary recalls. Plasma zonulin was used to evaluate intestinal permeability, while GM was determined by 16S rRNA sequencing. Regression models were used to analyze laboratory markers of lupus disease (C3 and C4 complement and C-reactive protein). Our results showed that the genus Megamonas was significantly enriched in the iSLE group (*p* < 0.001), with Megamonas funiformis associated with all evaluated laboratory tests (*p* < 0.05). Plasma zonulin was associated with C3 levels (*p* = 0.016), and sodium intake was negatively associated with C3 and C4 levels (*p* < 0.05). A combined model incorporating variables from each group (GM, intestinal permeability, and food intake) demonstrated a significant association with C3 complement levels (*p* < 0.01). These findings suggest that increased Megamonas funiformis abundance, elevated plasma zonulin, and higher sodium intake may contribute to reduced C3 complement levels in women with inactive SLE.

## 1. Introduction

Recent research has suggested that interactions between gut microbiota (GM) and the host immune system may contribute to the development of immune complex-mediated diseases in genetically susceptible individuals [1,2,3]. Studies have also indicated that specific bacterial taxa and metabolites may be associated with clinical and laboratory findings in patients with extra-intestinal autoimmune diseases, including Systemic Lupus Erythematosus (SLE) [3]. SLE is one of the most prevalent extra-intestinal autoimmune diseases and is characterized by the unregulated production of autoantibodies and immune-mediated tissue damage [4]. The cause of SLE is not fully understood, but genetic and environmental factors are thought to play a role [5,6,7].

Previous studies have reported on the association between SLE and GM, but the results have been controversial [8,9,10,11]. Some studies have suggested that changes in GM composition can influence the production of antinuclear antibodies and promote a toxic inflammatory micro-environment, leading to autoimmunity loss. However, the functional impact of GM on the development and progression of SLE still requires further investigation. Furthermore, it is unclear whether changes in GM composition in SLE are intrinsic characteristics of the disease or are modified in its remission phase [12,13].

In addition, the ingestion of macronutrients and specific micronutrients, such as sodium, vitamin D, and selenium, has been considered to modulate the development and complications of autoimmune diseases [14,15]. While the impact of food intake on GM is known [16], the interaction between GM, intestinal permeability, and food intake in SLE is not fully understood.

Therefore, the aim of this study was to investigate the relationship between GM, intestinal permeability, and food intake on inflammatory markers in inactive SLE patients. A better understanding of this interaction could help identify new biomarkers or therapeutic approaches for SLE.

## 2. Materials and Methods

### 2.1. Study Design and Subjects

This is a prospective, observational, and cross-sectional single-institution study approved by the local ethics committee (CaPPesq 3.829.175). Written informed consent was acquired from all participants before the beginning of the study and all protocol interventions were performed according to the Declaration of Helsinki guidelines.

Patients with inactive Systemic Lupus Erythematosus (iSLE) (22 females) and 20 healthy female volunteers were recruited. Inclusion criteria for iSLE patients were: female gender, aged over 18 years and under 51 years, fulfilled the 1997 revised criteria of the American College of Rheumatology for the diagnosis of SLE [17], were regular followed-up (at intervals of 1–6 months) by the Rheumatology Service of the Hospital das Clínicas, Faculdade de Medicina da Universidade de São Paulo (HC-FMUSP), disease activity assessed by Systemic Lupus Erythematosus Disease Activity Index 2000 (SLEDAI-2K) [18] with <3 score (defined as inactive or low disease activity) [19], and exclusively using the medication hydroxychloroquine (HCQ) on a stable dose for 4 weeks before enrollment in the study. Women who presented one or more of the following items were excluded: pregnancy or breastfeeding, menopause, a disease associated with proven modification of the GM signature (e.g.,: diabetes, metabolic disease, liver disease, malignant neoplasms, etc.), body mass index (BMI) less than 18.5 kg/m^2^ and greater than 30 kg/m^2^, renal impairment and/or acquired immunodeficiency syndrome (HIV) or known to be HIV positive; and use of one of the following drugs in the last 3 months: cyclophosphamide, antibiotics, corticosteroids, azathioprine, mycophenolate mofetil or methotrexate. Patients with low cognitive capacity, which would make adherence to the study difficult, were also excluded.

The inclusion criteria for Healthy Control (HC) group were as follows: women over 18 and under 51 years of age, with self-report of the absence of an acute and chronic clinical condition, without regular use of medication (except contraceptives). Women who presented one or more of the following items were excluded: pregnancy or breastfeeding; menopause; BMI less than 18.5 kg/m^2^ and greater than 30 kg/m^2^; short-term postoperative period (3 months) of medium or large surgeries; having undergone bariatric surgery at any time before participating in the study; and drug or alcohol abuse.

The selected participants underwent the following data collection and samples: data for characterizing the population (sociodemographic, anthropometric, body composition, and clinical history of the disease), food intake, intestinal permeability, and GM.

### 2.2. Patient’s Characterizing Data

For both groups, a trained researcher applied a semi-structured questionnaire to collect sociodemographic data. The anthropometric assessment included weight and height measurements to calculate the BMI, determined by the individual’s weight divided by height squared and expressed in kg/m². Body composition was evaluated using a four-frequency (5, 50, 100, 200 kHz) portable electric bioimpedance device (Quadscan 4000, Bodystat Ltd.^®^, USA/Canada).

Exclusively for the iSLE, clinical information regarding the time since diagnosis of the disease (in years), period of disease remission (in years), SLEDAI-2K score, current HCQ dosage (mg/kg/day), and period of stable HCQ dose (in years).

### 2.3. Food Intake

Food intake data were obtained through three 24-h recalls on non-consecutive days, applied close to the stool collections. The recalls were completed by trained researchers in a structured and semi-directed interview with questions about the food intake, preparations, and quantity in household measures [20]. After standardization, the research team converted these units into grams or milliliters [21]. Energy, macronutrient, and total fiber intake were determined using the EasyDiet^®^ software (version 1.0.14), which includes the Brazilian Food Composition Table [22] and the Family Budget Survey [23].

Based on the cross-sectional population Health Survey of São Paulo (HS-SP), this study applied the daily intake of 21 food groups [24]: fruits and vegetables (all fruits and vegetables, raw or cooked, except for beans), juices (natural and industrialized), rice and beans (brown beans, black beans, cooked white rice), pasta (cooked noodles, spaghetti, gnocchi, lasagna, cannelloni, ravioli), roots and tubers (potato, sweet potato, and cassava), bread (French bread, Italian bread, loaf bread, buns, French toast, bagels, crackers), whole wheat bread reduced-calorie or not, red meat (hamburger, steak beef, ground beef, beef ribs, pork chop, pork ribs, pork loin), processed meat (sausages, frankfurters, nuggets, bacon, ham, mortadella, salami, roast beef), poultry (chicken, turkey), fish (cod, sardines, tuna, white fish, tilapia, salmon—fresh and canned), eggs (fried and cooked), cheese (white cheese, ricotta cheese, cream cheese, mozzarella cheese, parmesan cheese), milk (0% to 3% fat milk), yogurt (yogurts and fermented milk), butter and margarine (salted and unsalted), coffee and tea (all kinds of coffee and herbal tea), sodas and sport drinks (high- and low-energy soda and energy drinks), alcoholic beverages (beer, wine, distillates, and cocktails), sweets (cakes, sweet pies, pudding, typical Brazilian sweets, industrialized sweets, ice cream, cookies, chocolate—bars and powder—and white sugar, honey and jams), salty pastries and pizza (sandwiches, croissants, Italian focaccia, kebab, and pizzas).

The Multiple Source Method (MSM) was applied to estimate the usual intake of energy, nutrients, and food groups through the online platform [25]. All nutrients were adjusted for total energy intake by the residue method [26].

### 2.4. Intestinal Permeability

Assessment of intestinal permeability was performed in the iSLE group using plasma zonulin. For this, blood from participants of the iSLE group was collected to obtain plasma (EDTA tubes, centrifugation at 878× *g*, 4 °C, 10 min). Plasma zonulin concentrations were evaluated using a competitive immunoenzymatic assay (ELISA, Elabscience^®^ Biotechnology Inc., San Francisco, CA, USA).

### 2.5. Gut Microbiota (GM)

Participants were instructed to use a sterile swab to transfer a small amount of fecal sample (in an amount similar to a bean grain) into 2 mL plastic microtubes containing a DNA preservation buffer solution. GM assessments were carried out by obtaining fecal DNA and amplifying the V3 e V4 region of the 16S rRNA gene, as detailed in International Human Microbiome Standards (IHMS) SOP06 (http://www.microbiome-standards.org; 1 July 2021). To achieve better bacterial identification resolution, bioinformatic analysis of the 16S rRNA data was performed by analyzing amplicon sequence variants (ASV), in the Bioinformatics Platform of the Rene Rachou Institute, Fiocruz Minas (Belo Horizonte, MG, Brazil).

In this method, primers used in amplification were removed and sequences with more than 2 expected errors were discarded. The remaining sequences were used to train an error identification and correction model; and direct and reverse readings, already corrected, were concatenated to form ASVs, to remove chimeric sequences and quantify ASVs. Each ASV had its taxonomic classification assigned by the TAG.ME package [27] using the specific model for the amplicon that corresponds to the region V3 e V4. After, species-level taxonomic identifications were assigned to each ASV using DADA2 [28], based on exact correspondence between ASVs and the reference sequences in the Silva database (version 132) [29].

### 2.6. Outcomes

Lupus disease parameters included SLEDAI-2K score at entry and as outcome in statistical regression models. Levels of complement C3, C4, and C-reactive protein (CRP) were measured by immunoturbidimetry at the Central Laboratory of HC-FMUSP.

### 2.7. Statistical Analysis

Continuous variables are presented as mean and standard deviation and categorical variables are presented as absolute and relative frequencies. The normality of continuous variables was assessed using the Shapiro-Wilk test. Comparisons between groups of the anthropometric and body composition data, food intake variables, and plasma zonulin were performed by *t*-test or Mann-Whitney test.

Simpson, Shannon, and Chao1 indices for alpha (α) diversity were calculated using the vegan R package (version 2.5-7), while population beta (β) diversity was calculated by Jehnsen-Shannon divergence. The Permanova analysis with *p*-value adjusted by Benjamini-Hochberg was also performed to identify the significant difference between groups in the macrostructure of the bacterial population.

Associations between independent variables (GM, plasma zonulin, food intake) and variables related to markers of clinical manifestations (outcomes) were evaluated by simple and multiple linear regression. Therefore, independent variables that were significantly different between groups (*p* < 0.05) were separated and tested individually and as a group for the effect on disease markers. The best subset of predictors was estimated for each dependent variable. The selection of these subsets was based on the values of the F statistic, the greatest significance of the estimates, adjusted R2, mean squared error, Masllow’s Cp, and Akaike’s information criterion. After finding the best models, they were tested for normality, heteroscedasticity, multicollinearity, and autocorrelation.

For all analyses, specific packages of the R software (version 4.2) were used and a significance level of 5% was adopted (*p* < 0.05).

## 3. Results

### 3.1. Patient’s Descriptive Data

Forty-two women with a mean of 34.0 and 6.6 years of age were included in the study. The body composition of patients did not differ between groups (*p* > 0.05). Disease data and clinical manifestation markers indicate that all patients with SLE were in remission (Table 1).

### 3.2. Food Intake

iSLE patients had a lower intake of saturated fat, cholesterol, phosphorus, copper, manganese, niacin, vitamin A, and vitamin C (*p* < 0.05); and a higher intake of carbohydrates, sodium, vitamin B12, and folate (*p* < 0.05) when compared to healthy women (Table 2).

In the assessment of food groups, iSLE patients had lower consumption of fruits and vegetables, whole wheat bread, eggs, cheese, yogurt, coffee, and tea (*p* < 0.05); and higher intake of juices, rice and beans, pasta, processed meat, sweets, salty pastries, and pizza (*p* < 0.05) when compared to healthy women. No patient in the iSLE consumed fish, while in the healthy group, no patient consumed sodas and sports drinks (Table 3).

### 3.3. Gut Microbiota (GM)

No statistical difference was observed between groups in any alpha diversity parameters (Supplementary Figure S1). When analyzing beta diversity between groups, a difference of 2.44% was observed in the macrostructure of the bacterial population without statistical significance (R^2^ = 0.024; *p*-adjusted value = 0.439; Supplementary Figure S2).

**Figure 1:**
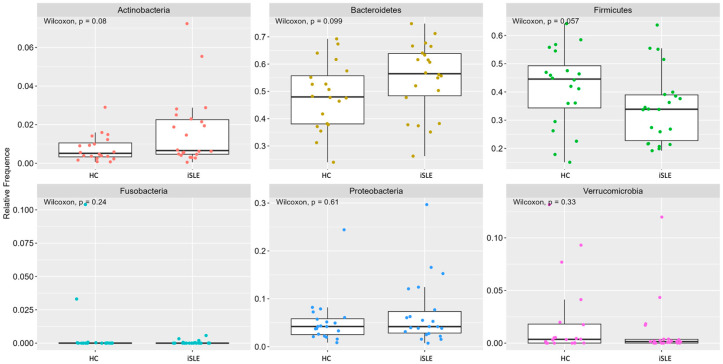
Relative abundance (%) of intestinal bacterial phyla in healthy control (HC; *n* = 20) versus inactive systemic lupus erythematosus group (iSLE; *n* = 22). Statistical significance *p* < 0.05.

**Figure 2:**
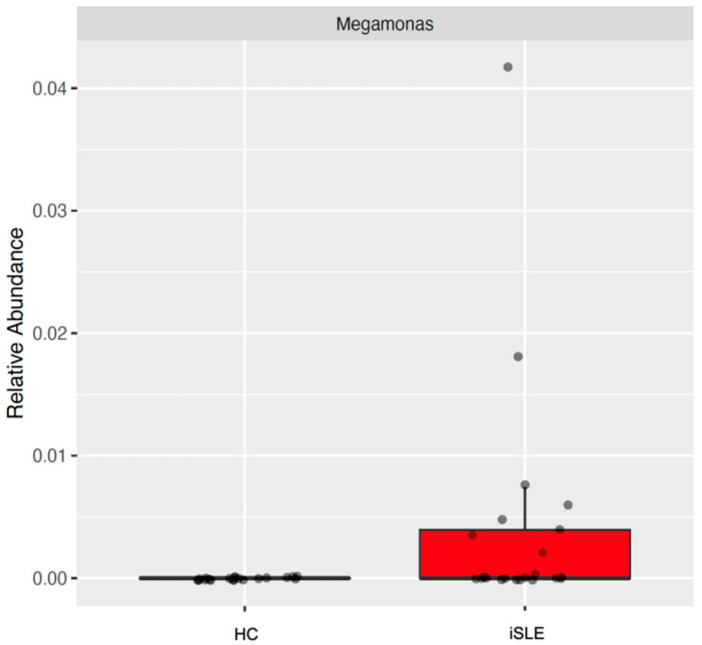
Relative abundance (%) of Megamonas in healthy control (HC; *n* = 20) versus inactive systemic lupus erythematosus (iSLE; *n* = 22). Statistical significance *p* < 0.05.

As shown in Figure 1, the most abundant phyla in the microbiota of healthy women and those with inactive SLE were *Bacteroidetes*, *Firmicutes*, *Actinobacteria*, *Proteobacteria*, *Verrucomicrobia*, and *Fusobacteria*, with no difference between groups. However, when assessing the differential abundance of genera and species between groups, we found the genus *Megamonas* significantly enriched in iSLE, with FoldChange 27.98 and *p* < 0.001 (Figure 2).

### 3.4. Regression Models

We investigated whether variables with a statistical difference between groups could significantly influence laboratory markers of lupus disease (C3 and C4 complement; CRP) by running a linear model (Supplementary Table S1). Megamonas funiformis was directly associated with all evaluated clinical manifestation markers (*p* < 0.05). Plasma zonulin was directly associated only with C3 complement [−0.80 (−0.30, 1.30); *p* = 0.016).

For food intake, sodium intake was negatively associated with C3 complement [−0.08 (−0.14, −0.02); *p* = 0.05] and C4 [−0.004 (−0.01, −0.001); *p* = 0.03], indicating that an increase in sodium intake is associated with a reduction in C3 and C4 complement levels. Higher intake of processed meats was also associated with reduced C4 [−0.38 (−0.61, −0.16); *p* = 0.02] and an increase in CRP [2.24 (0.71, 3.77); *p* = 0.03]. Furthermore, intake of saturated fat and cheese only increased CRP levels (*p* < 0.05; Supplementary Table S1), while whole-grain bread, fruits, and vegetables decreased CRP levels (*p* < 0.05; Supplementary Table S1).

A combined model incorporating variables from each group (GM, intestinal permeability, and food intake) demonstrated a significant association with C3 complement levels (Table 4). Together, increased Megamonas funiformis, plasma zonulin, and sodium intake affected C3 complement levels negatively (R^2^ 0.55; R^2^ adj 0.47; *p* < 0.01).

## 4. Discussion

In the present study, we have observed that the bacteria genus—*Megamonas*—was abundant in women with inactive SLE using HCQ but not in healthy women. In addition, our study demonstrated that *Megamonas funiformis* was associated with complement C3, C4, and CRP, and when associated with a leaky gut and higher sodium intake, it can negatively modulate C3 complement levels.

The association between complement reduction and SLE, particularly in active disease and infection, is well established [30]. However, the abundance of the *Megamonas* genus in inactive SLE patients using HCQ has not been previously reported. A recent study evaluated active SLE patients (score SLEDAI-2K = 16.16 ± 24.31 points) and reported a negative correlation between *Megamonas* abundance in the bladder microbiome and C3 complement levels. Notably, HCQ dosage did not affect microbial communities [31]. Conversely, gut *Megamonas* has been reported to be depleted in the gut of SLE patients and positively correlated with helper T cells 17 (Th17) [32]. In patients with primary Sjögren’s Syndrome, *Megamonas* was significantly more abundant than healthy controls and positively associated with clinical disease indicators [33]. Additionally, in populations with non-rheumatological diseases, *Megamonas* has been associated with pro-inflammatory cytokines [34]. These findings highlight the need for further investigation into the role of *Megamonas* in SLE pathology.

A significant finding in our study was the dietary intake of women with i-SLE. These women consumed higher amounts of carbohydrates, sodium, processed meats, and sweets, and lower amounts of fruits and vegetables, whole wheat bread, eggs, cheese, yogurt, and certain vitamins and minerals (phosphorus, copper, manganese, niacin, vitamin A, and vitamin C) compared to healthy women. While the role of lifestyle factors in SLE remains controversial, dietary habits and their impact on the microbiome composition are receiving more attention from researchers [35,36].

It is known that a western dietary pattern, characterized by a high intake of processed foods, refined grains, sugar, and sodium, and a low intake of fruits, vegetables, legumes, and whole grains, may contribute to the risk, pathophysiology, and management of autoimmune diseases [14]. Our findings add to this growing body of evidence, highlighting the potential role of dietary factors in SLE.

Our study draws attention to the connection of some of these components with laboratory markers of lupus disease and inflammation, with emphasis on the higher intake of processed meat associated with increased CRP and decrease in C4 complement levels; and the higher intake of whole grains, fruits, and vegetables associated with a decrease in CRP levels.

In terms of sodium intake, ingestion appears to have negative effects on patients with SLE. Sodium chloride is known to induce the polarization of macrophages towards pro-inflammatory phenotypes and promote Th17-mediated pro-inflammatory immune responses [37]. Additionally, increased levels of sodium in skeletal muscle tissue have been reported in SLE patients, and this has been associated with disease activity as measured by SLEDAI-2K scores [38]. In a murine model of SLE, a high-salt diet has been shown to significantly exacerbate the disease [39]. Furthermore, restricted sodium intake in SLE patients has been linked to a decrease in Th17 cells and an increase in regulatory T cells, suggesting that a low-sodium diet may help alleviate the inflammatory response in SLE [40]. Our findings are consistent with these results, as variations in sodium intake were found to be inversely correlated with C3 and C4 complement levels.

Dietary components, both micronutrients, and macronutrients, have been shown to affect various innate physical defenses, such as epithelial barrier integrity, antimicrobial peptides, pro/anti-inflammatory cytokines, and immune cell functionality [41,42,43]. In vitro studies have demonstrated that certain dietary components can impair human epithelial barrier function, leading to increased intestinal permeability across tight junctions. This results in the passage of toxins, food antigens, and bacteria, some of which may carry immunogenic antigens [44]. Furthermore, a lack of dietary fiber from fruits, vegetables, legumes, and whole grains, which are not commonly consumed in sufficient amounts, may deprive the intestinal microbiota of necessary nutrients, leading to the induction of enzymes capable of degrading the intestinal mucin layer, ultimately contributing to the development of a leaky gut [15,44].

Growing evidence suggests that some, if not all, SLE patients may experience a leaky gut [45,46]. The detection of microbial components in the bloodstream of SLE patients further suggests that increased intestinal permeability may mediate the penetration of microorganisms and their products into the systemic circulation [47,48,49]. It is hypothesized that leaky gut, overactive immune responses, and microbial dysbiosis may exacerbate lupus disease, creating a vicious feed-forward loop [50]. Our study supports this hypothesis, as higher plasma zonulin concentrations were associated with lower C3 complement levels and a tendency towards increased CRP levels. Furthermore, our findings indicate that dietary factors, including GM, intestinal permeability, and food intake, can affect C3 complement levels.

This study aimed to demonstrate that even in i-SLE patients, changes in GM composition, intestinal permeability, and food intake may be associated with fluctuations in laboratory markers. We hypothesized that a pre-phase with low-grade inflammation might precede the clinical manifestation of SLE. Despite the limitations of this study, such as the small sample size and recruitment from a single center, strict criteria were applied to select patients, resulting in a homogeneous sample. Further studies measuring plasma complement breakdown products and cell-bound activation products are encouraged to validate our results.

## 5. Conclusions

In our study, we observed that women with inactive SLE on HCQ treatment exhibited a significantly greater prevalence of *Megamonas* in their gut microbiome, and a diet rich in carbohydrates, sodium, vitamin B12, and folate, when compared to healthy women. The elevated presence of *Megamonas funiformis*, together with heightened intestinal permeability and excessive sodium consumption, adversely affects C3 complement levels. Consequently, implementing adjuvant therapies targeting the reduction of these factors could prove to be a valuable approach in extending the duration of remission in SLE patients

## Figures and Tables

**Table 1:** Characterization of participants according to anthropometric, body composition, clinical and biochemical variables.

Variables	iSLE ^1^ (*n* = 22)	HC ^2^ (*n* = 20)	*p*-Value ^3^
Age, years	36.23 ± 6.17	31.55 ± 6.48	0.013
Gender			
Female, *n* (%)	22 (100)	20 (100)	1.0
Masculine, *n* (%)	0 (0)	0 (0)	
Anthropometric data			
Weight, kg	66.48 ± 8.08	60.75 ± 8.35	0.015
Heigh, cm	163 ± 0.08	164 ± 0.05	0.811
BMI ^4^, kg/m^2^	24.9 ± 2.7	22.6 ± 2.6	0.015
Body Composition data			
LMW ^5^, kg	43.6 ± 5.4	42.4 ± 4.0	0.272
LMP ^6^, %	66.4 ± 5.8	68.7 ± 5.1	0.182
FMW ^7^, kg	22.3 ± 5.2	19.7 ± 5.3	0.123
FMP ^8^, %	33.6 ± 5.8	31.4 ± 5.1	0.182
Disease data			
Time of ilness, years	12.5 ± 6.4	NA ^9^	
Time Remission, years	4.2 ± 5.4	NA ^9^	
SLEDAI-2K score ^10^	0.0 ± 0.4	NA ^9^	
Medication data			
HCQ ^11^, mg/kg/day	4.7 ± 0.6	NA ^9^	
SDT-HCQ ^12^, years	2.5 ± 1.4	NA ^9^	
Laboratorial parameters			
C3 Complement, mg/dL	99.1 ± 18.2	NA ^9^	
C4 Complement, mg/dL	17.3 ± 5.4	NA ^9^	
C-reactive protein, mg/dL	1.8 ± 1.9	NA ^9^	
Intestinal Permeability			
Plasma zonulin, ng/mL	40.3 ± 11.5	NA ^9^	

^1^ iSLEG = inactive Systemic Lupus Erythematosus. ^2^ HC = Healthy Control. ^3^ *t*-test or Mann-Whitney test. ^4^ BMI = Body Mass Index. ^5^ LMW = Lean Mass Weight. ^6^ LMP = Lean Mass Percentage. ^7^ FMW = Fat Mass Weight. ^8^ FMP = Fat Mass Percentage. ^9^ NA = not applicable. ^10^ SLEDAI-2k = Systemic Lupus Erythematosus Disease Activity Index. ^11^ HCQ = Hydroxychloroquine. ^12^ SDT-HCQ = Stable Dose Time—Hydroxychloroquine. Statistical significance *p* < 0.05.

**Table 2:** Daily intake means of macronutrients and micronutrients of participants.

Variables	iSLE ^1^ (*n* = 22)	HC ^2^ (*n* = 20)	*p*-Value ^3^
Energy, kcal	1753.7 ± 18.6	1593.9 ± 338.1	0.116
Carbohydrates, g	222.4 ± 63.7	177.5 ± 44.1	0.004
Protein, g	72.5 ± 11.0	77.5 ± 18.6	0.609
Total fat, g	62.8 ± 3.5	63.0 ± 3.5	0.541
Saturated fat, g	20.1 ± 0.2	22.9 ± 3.2	0.005
Monounsaturated fat, g	17.6 ± 0.3	19.1 ± 5.2	0.284
Polyunsaturated fat, g	14.3 ± 3.5	13.0 ± 2.7	0.268
Cholesterol, mg	246.4 ± 62.7	316.1 ± 59.4	<0.001
Fiber, g	16.8 ± 2.8	17.8 ± 4.3	0.529
Sodium, mg	3379.6 ± 742.5	2762.0 ± 589.3	0.006
Calcium, mg	604.2 ± 174.5	683.6 ± 121.7	0.085
Iron, mg	8.2 ± 1.5	8.2 ± 1.9	0.930
Magnesium, mg	218.5 ± 75.2	238.0 ± 83.9	0.509
Selenium, µg	19.0 ± 0.3	19.2 ± 2.9	0.124
Phosphorus, mg	435.4 ± 101.1	482.5 ± 75.0	0.050
Copper, µg	0.4 ± 0.2	0.7 ± 0.2	<0.001
Manganese, mg	0.3 ± 0.1	1.2 ± 0.3	<0.001
Potassium, mg	2017.2 ± 510.0	2206.9 ± 725.0	0.525
Zinc, mg	8.7 ± 2.3	8.5 ± 2.1	0.681
Thiamine, mg	0.9 ± 0.0	0.8 ± 0.2	0.301
Niacin, mg	3.9 ± 0.7	5.9 ± 2.5	<0.001
Pyridoxxine, mg	0.6 ± 0.4	0.5 ± 0.1	0.199
Vitamin B12, µg	1.5 ± 0.6	1.1 ± 0.3	0.022
Vitamin A, µg	194.6 ± 73.1	264.9 ± 80.7	0.010
Vitamin C, mg	77.3 ± 27.4	123.8 ± 49.7	<0.001
Vitamin D, µg	1.5 ± 0.7	1.4 ± 0.5	0.488
Vitamin E, mg	1.8 ± 0.0	1.6 ± 0.4	0.434
Folate, µg	81.1 ± 13.7	66.2 ± 15.6	<0.001

^1^ iSLE = inactive Systemic Lupus Erythematosus. ^2^ HC = Healthy Control. ^3^ *t*-test or Mann-Whitney test. Statistical significance *p* < 0.05. Data are shown as the mean ± standard deviation.

**Table 3:** Daily intake means of food groups of participants.

Variables	iSLE ^1^ (*n* = 22)	HC ^2^ (*n* = 20)	*p*-Value ^3^
Fruits and vegetables, g	188.1 ± 95.4	291.0 ± 103.2	0.002
Juices, mL	123.6 ± 61.1	84.9 ± 71.2	0.042
Rice and beans, g	182.5 ± 78.8	123.2 ± 86.9	0.026
Pasta, g	48.3 ± 42.3	28.3 ± 40.9	0.023
Roots and tubers, g	68.8 ± 66.3	50.8 ± 38.1	0.753
Bread, g	49.9 ± 40.8	22.9 ± 11.3	0.129
Whole wheat bread, g	2.6 ± 8.0	17.8 ± 17.5	<0.001
Red meat, g	61.7 ± 31.3	54.4 ± 40.1	0.336
Processed meat, g	21.6 ± 7.5	7.7 ± 6.5	<0.001
Poultry, g	50.7 ± 27.8	59.3 ± 19.5	0.260
Fish, g	0.0 ± 0.0	15.5 ± 10.4	NA ^4^
Eggs, g	9.3 ± 13.6	33.9 ± 11.4	<0.001
Cheese, g	15.0 ± 9.7	39.0 ± 12.9	<0.001
Milk, mL	110.5 ± 110.9	85.3 ± 77.3	0.288
Yogurt, g	18.3 ± 49.1	21.7 ± 27.2	<0.001
Butter and margarine, g	3.7 ± 4.4	3.7 ± 3.6	0.465
Coffe and tea, mL	107.1 ± 76.1	176.4 ± 81.2	0.007
Sodas and sport drinks, mL	115.0 ± 81.0	0.0 ± 0.0	NA ^4^
Alcoholic beverages, g	33.2 ± 64.5	76.0 ± 116.7	0.187
Sweets, g	103.2 ± 37.5	63.2 ± 30.8	<0.001
Salty pastries and pizza, g	39.3 ± 28.4	21.1 ± 14.7	0.025

^1^ iSLE = inactive Systemic Lupus Erythematosus. ^2^ HC = Healthy Control. ^3^ *t*-test or Mann-Whitney test. ^4^ NA = not applicable. Statistical significance *p* < 0.05. Data are shown as the mean ± standard deviation.

**Table 4:** Multiple regression model with the effect of gut microbiota, intestinal permeability, and food intake (independent variables) on C3 complement (dependent variable).

Variables	C3 ^1^
Gut Microbiota	
*Megamonas funiformis*	*−*128.33 (*−*45.01, *−*211.64) *p* = 0.03
Intestinal Permeability	
Plasma zonulin (ng/mL)	*−*0.63 (*−*0.20, 1.05) *p* = 0.03
Food intake	
Sodium (mg)	*−*0.06 (*−*0.10, *−*0.01) *p* = 0.07

^1^ Multivariate Regression Model: C3 R^2^ 0.55; R^2^ adj 0.47 (*p* < 0.01). Statistical significance *p* < 0.05.

## Supplementary Materials

The following supporting information can be downloaded at: https://www.mdpi.com/article/10.3390/nu15081999/s1, Supplementary Figure S1: Comparison of bacterial alpha diversity represented by (A) Shannon Index, (B) Simpson Index, and (C) Chao1 Index of the Healthy Control group (HC; n = 20) versus inactive Systemic Lupus Erythematosus (iSLE; n = 22). Supplementary Figure S2: Principal coordinate analysis of the gut microbiota macrostructure, based on the Jensen-Shannon index, comparing the Healthy Control group (HC) versus inactive Systemic Lupus Erythematosus (iSLE). Supplementary Table S1: Simple linear regression models for effects of variation of gut microbiota, plasma zonulin, and food intake (independent variables) in the manifestation markers of lupus disease—complement C3, C4, and C-reactive protein (dependent variables).
