# Supplementary material for: Megamonas funiformis, Plasma Zonulin, and Sodium Intake Affect C3 Complement Levels in Inactive Systemic Lupus Erythematosus
Source: Nutrients. 2023 Apr 21;15(8):1999. doi: 10.3390/nu15081999 (PMC10144636; doi:10.3390/nu15081999)
Supplement: Supplementary file 1 [file nutrients-15-01999-s001.zip › nutrients-2334021-supplementary.pdf]

nutrients

MDPI

Supplementary Materials

Megamonas funiformis, plasma zonulin, and sodium intake affect C3 complement levels in inactive Systemic Lupus Erythematosus

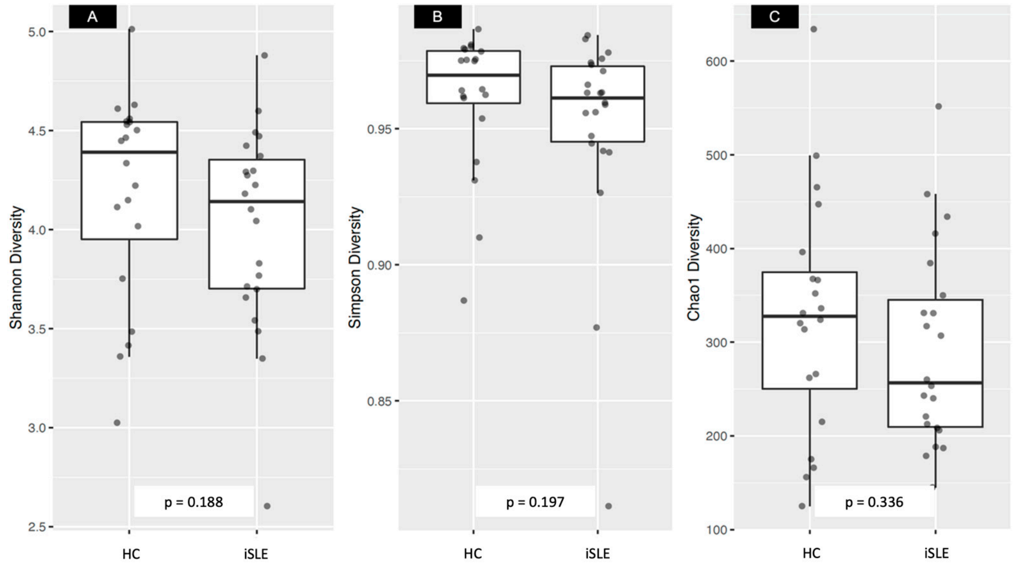

Supplementary Figure S1. Comparison of bacterial alpha diversity represented by (A) Shannon Index, (B) Simpson Index and (C) Chao1 Index of the Healthy Control group (HC; n = 20) versus inactive Systemic Lupus Erythematosus (iSLE; n = 22).

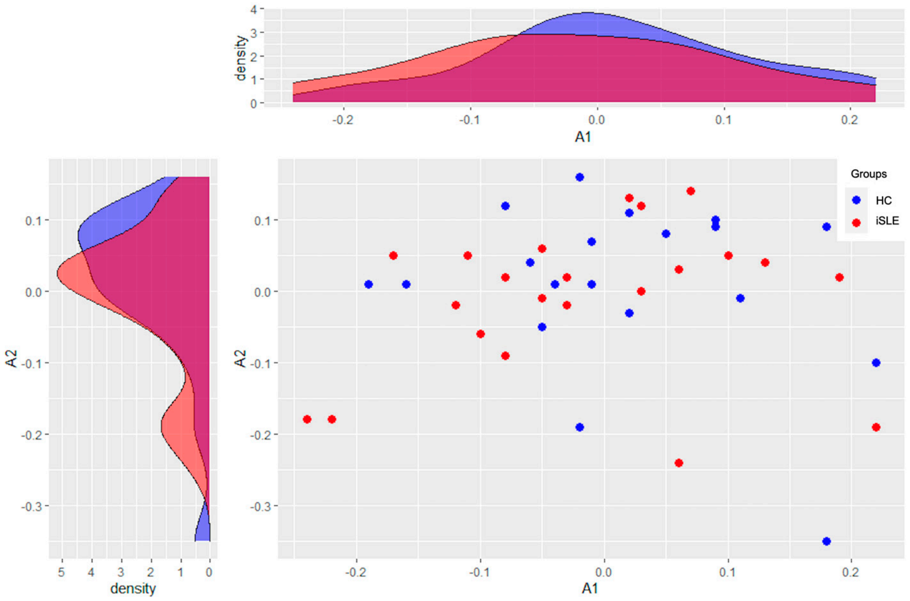

Supplementary Figure S2. Principal coordinate analysis of the gut microbiota macrostructure, based on the Jensen-Shannon index, comparing Healthy Control group (HC) versus inactive Systemic Lupus Erythematosus (iSLE).

nutrients

Supplementary Table S1. Simple linear regression models for effects of variation of gut microbiota, plasma zonulin, and food intake (independent variables) in the manifestation markers of lupus disease - complement C3, C4 and C-reactive protein (dependent variables).

Variables	C3 [1]	C4 [1]	CRP [1]
**Gut Microbiota**			
Megamonas funiformis	–115.72 (–176.42, –55.03)<br>**p = 0.01**	45.27 (10.04, 80.50)<br>**p = 0.05**	26.97 (20.66, 33.29)<br>**p < 0.001**
**Intestinal Permeability**			
Plasma zonulin (ng/mL)	–0.80 (–0.30, 1.30)<br>**p = 0.01**	0.18 (0.02, 0.33)<br>p = 0.08	0.06 (0.01, 0.12)<br>p = 0.07
**Food intake**			
Carbohydrates (g)	–0.10 (–0.20, –0.01)<br>p = 0.10	–0.03 (–0.06, –0.0004)<br>p = 0.12	–0.004 (–0.01, 0.01)<br>p = 0.54
Saturated Fat (g)	18.70 (–12.69, 50.10)<br>p = 0.34	4.91 (–4.36, 14.18)<br>p = 0.40	5.61 (3.04, 8.17)<br>**p = 0.002**
Cholesterol (mg)	0.03 (–0.08, 0.13)<br>p = 0.71	–0.03 (–0.06, 0.005)<br>p = 0.18	0.01 (–0.005, 0.02)<br>p = 0.38
Sodium (mg)	–0.08 (–0.14, -0.02)<br>**p = 0.05**	–0.004 (–0.01, –0.001)<br>**p = 0.03**	–0.001 (–0.002, 0.0003)<br>p = 0.26
Phosphorus (mg)	–0.01 (–0.02, -0.0000)<br>p = 0.12	–0.01 (–0.03, 0.01)<br>p = 0.38	–0.001 (–0.01, 0.01)<br>p = 0.84
Copper (ug)	–10.77 (–33.63, 12.09)<br>p = 0.45	4.29 (–2.34, 10.92)<br>p = 0.31	–0.88 (–3.23, 1.47)<br>p = 0.55
Manganese (mg)	–18.43 (–61.54, 24.68)<br>p = 0.50	3.01 (–9.76, 15.79)<br>p = 0.71	–2.83 (–7.17, 1.52)<br>p = 0.30
Niacin (mg)	0.22 (–8.38, 8.82)<br>p = 0.97	–0.24 (–2.77, 2.28)<br>p = 0.88	0.38 (–0.49, 1.25)<br>p = 0.48
Vitamin B12 (ug)	–9.37 (–20.12, 1.37)<br>p = 0.17	–0.69 (–4.00, 2.61)<br>p = 0.74	–0.20 (–1.36, 0.95)<br>p = 0.78
Vitamin A (ug)	–0.01 (–0.10, 0.08)<br>p = 0.89	0.02 (–0.004, 0.05)<br>p = 0.18	0.005 (–0.004, 0.01)<br>p = 0.40

Vitamin C (mg)	–0.08 (–0.32, 0.17)<br>p = 0.62	0.03 (–0.05, 0.10)<br>p = 0.57	–0.01 (–0.03, 0.02)<br>p = 0.70
Folate (ug)	–0.35 (–0.82, 0.12)<br>p = 0.25	–0.10 (–0.24, 0.04)<br>p = 0.25	–0.09 (–0.12, –0.05)<br>**p = 0.002**
Fruits and vegetables (g)	–0.01 (–0.08, 0.06)<br>p = 0.74	0.01 (–0.02, 0.03)<br>p = 0.69	–0.001 (–0.01, 0.01)<br>p = 0.74
Juices (ml)	–0.05 (–0.15, 0.06)<br>p = 0.51	0.01 (–0.02, 0.04)<br>p = 0.55	–0.005 (–0.02, 0.01)<br>p = 0.50
Rice and Beans (g)	0.01 (–0.08, 0.09)<br>p = 0.88	–0.03 (–0.05, –0.002)<br>p = 0.10	–0.002 (–0.01, 0.01)<br>p = 0.75
Pasta (g)	–0.14 (–0.29, 0.01)<br>p = 0.14	–0.03 (–0.07, 0.02)<br>p = 0.37	–0.02 (–0.03, –0.002)<br>p = 0.08
Whole wheat bread (g)	15.42 (–0.61, 31.45)<br>p = 0.13	4.17 (–0.59, 8.92)<br>p = 0.17	–0.15 (–0.22, –0.08)<br>**p = 0.004**
Processed Meat (g)	-0.88 (–1.71, –0.04)<br>p = 0.11	–0.38 (–0.61, –0.16)<br>**p = 0.02**	2.24 (0.71, 3.77)<br>**p = 0.03**
Eggs (g)	–0.04 (–0.53, 0.46)<br>p = 0.91	–0.03 (–0.18, 0.11)<br>p = 0.71	0.02 (–0.03, 0.07)<br>p = 0.53
Cheese (g)	–0.06 (–0.75, 0.63)<br>p = 0.90	0.12 (–0.08, 0.32)<br>p = 0.34	0.10 (0.04, 0.16)<br>**p = 0.02**
Yogurt (g)	–4.02 (–23.07, 15.04)<br>p = 0.74	3.27 (–2.21, 8.76)<br>p = 0.34	–0.70 (–2.64, 1.23)<br>p = 0.56
Coffee and Tea (ml)	0.01 (–0.08, 0.09)<br>p = 0.92	0.02 (–0.004, 0.05)<br>p = 0.18	0.003 (–0.01, 0.01)<br>p = 0.63
Sodas and Sport Drinks (g)	0.06 (–0.02, 0.14)<br>p = 0.25	–0.005 (–0.03, 0.02)<br>p = 0.75	0.002 (–0.01, 0.01)<br>p = 0.71
Sweets (g)	–0.04 (–0.22, 0.14)<br>p = 0.72	–0.02 (–0.07, 0.04)<br>p = 0.61	–0.02 (–0.03, 0.001)<br>p = 0.13
Salty Pastries and Pizza (g)	0.13 (–0.10, 0.36)<br>p = 0.36	–0.01 (–0.08, 0.06)<br>p = 0.83	0.01 (–0.02, 0.03)<br>p = 0.62

[1] Univariate Regression 18
